# Albuminuria and its correlates in an Iranian type 2 diabetic population

**DOI:** 10.1186/1476-511X-7-28

**Published:** 2008-08-10

**Authors:** Manouchehr Nakhjavani, Alireza Esteghamati, Fatemeh Esfahanian, Naser Aghamohammadzadeh, Sepehr Hamidi, Alipasha Meysamie, Mehrshad Abbasi

**Affiliations:** 1 Endocrine and Metabolism Research Center, Valiasr Hospital, Tehran University of Medical Sciences, Tehran, Iran; 2 Division of Internal Medicine, Tabriz University of Medical Sciences, Tabriz, Iran; 3 Department of Community and Preventive Medicine, Faculty of Medicine, Tehran University of Medical Sciences, Tehran, Iran

## Abstract

**Objective:**

To study the prevalence and correlates of increased urinary albumin excretion (UAE) in an Iranian type 2 diabetic population.

**Methods:**

Over a one year period since October 2002, 400 consecutive type 2 diabetic patients referred to an outpatient diabetes clinic, were enrolled in a cross sectional study. Subjects had no history of renal impairment or overt proteinuria. Data concerning demographic characteristics and cardiovascular risk factors were recorded and height, weight and blood pressure were measured. Glucose, cholesterol, HDL-C, LDL-C, triglyceride, apoprotein B, lipoprotein a, creatinine, and HbA1c were measured in fasting blood samples. Overnight twelve-hour UAE were assessed by immunoturbidometry method. Regression analyses were employed to determine the correlates of UAE.

**Results:**

Out of 400 patients, 156 (40%) subjects had increased UAE (UAE ≥ 30 mg/24 hour). The UAE was higher in males compared to females (145.5 vs. 72.1 mg/day; p < 0.05); however, the age and HDL adjusted UAE levels were not significantly different between men and women (120.1 vs. and 87.9 mg/day; p = 0.37). Increased UAE was correlated with decreasing HDL-C and a longer duration of diabetes independent of other variables; increased UAE was correlated with HbA1c as well. Age, systolic and diastolic blood pressure, total cholesterol, LDL-C, triglyceride, apoprotein B, lipoprotein a, and GFR did not correlate with increased UAE.

**Conclusion:**

In this study, increased UAE was considerably frequent among type 2 diabetic patients without any significant history of renal dysfunction. Albuminuria was found to be associated with dyslipidemia (low HDL-C), long duration of diabetes, and uncontrolled glycemia revealed by higher HbA1c.

## Background

The prevalence of type 2 diabetes (T2D) and its complications are on the rise in developing countries [1-3]. Increased urinary albumin excretion (UAE) is an indicator of damage of the vascular endothelium, an unyielding marker of cardiovascular disease pathologies [4,5] and renal impairment [6], and a strong marker of developing diabetes complications [1]. In Iran as a developing nation, diabetes is noticeably common (7.7% of 25–64 year old population) [8] and the association of diabetes and acute coronary events are well underscored [9]. Nevertheless, the prevalence and correlates of increased UAE have not been studied in Iranian diabetic patients so far. The intent of the present study was thus to study the prevalence of albuminuria and its correlates in an Iranian type 2 diabetic population.

## Methods

An uncontrolled registry single centre cross sectional study was conducted from October 2002 to September 2003 in the outpatient diabetes clinic of Vali-Asr Hospital, a teaching university hospital of Tehran University of Medical sciences (Tehran, Iran). Patients with T2D visited by two of the authors (M.N. and N.A.) were invited to participate in this study. Patients with elevated serum creatinine (1.3 mg/dl for women and 1.5 mg/dl for men), overt proteinuria revealed by dipstick, those with evidence of non-diabetic renal disease, heart failure, systemic or local infections, and those with FBS > 400 mg/dl were not included. Records of patients were reviewed and the diagnosis of T2D was established according to the criteria of the American Diabetes Association [10]. Health characteristics and demographic data of patients, including age, duration of diabetes and smoking were obtained by data sheet review. Height, weight in light clothing, and blood pressure (in sitting position after 10 minutes rest) were measured. The study protocol was approved by the review board of Endocrine Research Council and medical ethics committee of Tehran University of Medical sciences. All subjects gave informed consent.

Out of 435 visited eligible type 2 diabetic patients, 400 individuals participated in the biochemical examination and completed the study with valid UAE measurements. Twelve hour fasting blood samples of the patients were collected within one month of the visit. The patients were instructed to collect their urine in acid boric supplied containers from 7 pm to 7 am under standard conditions. Glucose, creatinine, triglyceride, total cholesterol, high-density lipoprotein cholesterol (HDL-C), low-density lipoprotein cholesterol (LDL-C), apoprotein B [apo(B)], and lipoprotein a [Lp(a)] were determined in blood samples and urine analyses were performed. Glycated hemoglobin (HbA1c) was determined in blood samples since June 2003 (122 patients). HDL-C, apo(B), and triglyceride were determined using immunoinhibition, immunoturbidometry, and Tinder enzymatic methods, respectively. UAE was measured by latex turbidimetric immunoassay method employing DAKO (Glostrop, Denmark) package.

### Definition of variables

Obesity was defined as body mass index (BMI, weight divided by square of height) equal or more than 30 kg/m^2 ^[11]. Hypertension was defined as the use of antihypertensive medication(s) or systolic blood pressure (SBP) of ≥ 140 mmHg or diastolic blood pressure (DBP) of ≥ 90 mmHg [12]. Smoking was defined as a history of regular daily smoking equal or more than one pack-year. Subjects with UAE > 30 mg/24 h were defined as having abnormal UAE or albuminuria. Normo-, micro-, and macro-albuminuria were defined as UAE < 30 mg/24 h, between 30–300 mg/24 h, and >300 mg/24 h. Twelve-hour overnight UAE level was doubled as an estimate of 24 hour UAE. Dyslipidemia was defined as LDL-C > 100 mg/dl, HDL-C < 40 mg/dl for men and <50 mg/dl for women, triglyceride > 150 mg/dl, apo (B) > 110 mg/dl, or Lp(a) > 30 mg/dl. Glomerular filtration rate (GFR) was calculated based on the Cockcroft-Gault formula estimated creatinine clearance.

### Statistical methods

Health characteristics of subjects with normal and abnormal UAE were compared with Chi-square analysis for qualitative data and Student's t-test for quantitative data, wherever appropriate. A series of bivariate regression analyses were used to examine the association of abnormal albuminuria with different independent variables including age, sex, duration of diabetes, BMI, glycated hemoglobin, triglyceride, total cholesterol, LDL-C, HDL-C, apo(B), and Lp(a). Mutivariate linear and binary (increased vs. normal UAE) regression models were designed to examine the correlates of abnormal albuminuria employing significant correlated variables of bivariate regression analyses. The univariate general linear model was applied to perform regression analysis and analysis of variances for UAE levels – as dependent variable – by sex as fixed factor, and age, duration of diabetes, and HDL-C as co-variables. Likewise, the univariate general linear models were employed to examine the equality of HDL-C among different albuminuria and sex strata with the confounding variables as covariates including age and duration of diabetes. A probability level of < 0.05 was considered statistically significant. Data was analyzed using SPSS Version 13.0 for Windows (SPSS, Chicago, IL).

## Results

Out of 400 (116 males and 284 females) diabetic patients, 133 individuals had microalbuminuria (33%; 95% confidence intervals (CI): 28–38%), and 23 had macroalbuminuria (5.8%; CI: 3.5–8%). Subjects with abnormal UAE had lower HDL-C (42.6 ± 13.5 vs.46.1 ± 16.1 mg/dl; p < 0.05), higher triglyceride (225.1 ± 139.3 vs. 199.2 ± 116.6 mg/dl; p = 0.055) and longer durations of diabetes (10.8 ± 8 vs. 7.7 ± 6.8 years; p < 0.001). Age, BMI, SBP and DBP, and serum lipids including LDL and total cholesterol, apo(B) and Lp(a) were not different between subjects with normal and abnormal UAE (table 1). In females, differences of HDL-C, triglyceride, and duration of diabetes between subjects with normal and abnormal albuminuria were found to be significant. In males, the difference of duration of diabetes between those with abnormal and normal UAE was significant.

**Table 1:** Health characteristics of participants in respect to gender and urinary albumin excretion

	Males (n = 116)		Females (n = 284)		Total		
	Normal UAE (n = 57)	Abnormal UAE (n = 59)	Normal UAE (n = 187)	Abnormal UAE (n = 97)	Normal UAE (n = 244)	Abnormal UAE (n = 165)	All participants (n = 400)
Age	56.5(12.6)	57.3(10.8)	52.8(10.4)	54.7(10.3)	53.6(11)	55.6(10.5)	54.4(10.9)
duration of diabetes(yr)	7.2(6.4)	12(9.3) †	7.8(6.9)	10.1(7.2) †	7.7(6.8)	10.8(8)†	8.9(7.4)
Systolic BP	126.9(17.1)	131.2(13.9)	132.4(21.7)	135(21.7)	131.2(20.8)	133.6(19.2)	132.1(20.2)
Diastolic BP	75.7(11.7)	79.8(9.5)	80.9(11.5)	82.2(11.9)	79.7(11.7)	81.3(11.1)	80.3(11.5)
GFR (calculated)	90.3(39.2)	89.4(33.1)	94.2(29.3)	88.3(39.4)	93.5(31.1)	88.7(37.3)	91.7(33.6)
Cholesterol	193.4(45.7)	188(60.2)	231.9(70.5)	230(63.3)	223.1(67.5)	213.9(65.2)	219.4(66.7)
HDL-C	39.9(11.7)	39.6(16.6)	48(16.8)	44.4(10.9) †	46.1(16.1)	42.6(13.5) †	44.7(15.2)
LDL-C	120.1(35.7)	116.5(43.5)	141.3(47.6)	135.8(46.1) †	136.2(45.9)	128.1(45.9)	133(46)
Triglycerides	166.1(75.9)	180.9(106.5)	209.2(124.8)	252.6(150.4)	199.2(116.6)	225.1(139.3)	209.5(126.6)
APOB	101.9(23.2)	97.6(29.8)	114.9(32)	118.5(29.7)	111.9(30.7)	110.4(31.4)	111.3(30.9)
Lp(a)	45.6(37.8)	38.3(37.2)	48.5(37.3)	59.8(52.4)	47.8(37.4)	51.4(48.1)	49.2(41.9)
FPG	222.5(81.1)	191.9(70.6)	194(70.4)	214.9(94.8)	199.7(73.3)	206.5(87.1)	202.3(78.8)
HbA1c	7.8(2.1)	9.5(2.5)	9.2(2.8)	10(3.6)	8.9(2.7)	9.8(3.2)	9.3(3)

Data are mean (standard deviation); † indicates p < 0.05; BP: blood pressure; FPG: fasting plasma glucose; GFR: glomerular filtration rate

The frequency of increased UAE was significantly higher in men than in women (51% vs. 34%, p < 0.005). Male participants were older than females (56.9 ± 11.7 vs. 53.4 ± 10.4 years, p < 0.05) but the duration of diabetes was the same in males and females (9.5 ± 8.3 vs. 8.6 ± 7.1 years). Women had higher mean BMI, DBP, total cholesterol, LDL-C, HDL-C, triglyceride, apo(B), and Lp(a) compared to men (data not shown). The mean UAE value was higher in men compared to women (145.5 vs. 72.1 mg/day; p < 0.05); however, age adjusted UAE of men was not significantly higher than that of women (134.4 mg/day (95% confidence intervals: 78.6–190.2) vs. 77.8 mg/day (42.8–112.8); p = .09); likewise, age and HDL-C adjusted UAE values were respectively 120.1 mg/day (CI: 61.2–179) and 87.9 mg/day (CI: 51.1–124.7) in males and females (p = 0.37).

Results of regression analyses are shown in table 2. In bivariate analyses, UAE was correlated with HDL-C (r = -0.15; p < 0.001), duration of diabetes (r = 0.13; p < 0.05), and HbA1c (r = -0.24, p < 0.001). UAE had no significant correlation with age, BMI, SBP and DBP, blood glucose level, total cholesterol, LDL-C, triglyceride, apo(B), Lp(a), and GFR. None of the above mentioned variables was found to have significant correlation with UAE in males. In females, duration of diabetes (r = 0.2; p < 0.005), HDL-C (r = 0.16; p < 0.05), Lp(a) (r = 0.14; p < 0.05), and HbA1c (r = 0.3; p < 0.005) correlated with UAE. Two models including sex, duration of diabetes, HDL-C, and Lp(a) with and without HbA1c were designed for multivariate analyses of the correlates of abnormal UAE. In first model, duration of diabetes and HDL-C levels were associated with UAE (P < 0.005). In second multivariate regression model, HDL-C and HbA1c were associated with UAE (P < 0.01).

**Table 2:** Correlates of increased urinary albumin excretion

	Binary logistic regression		Linear Regression		
	EXP(B)	95% CI for EX9B)	sig	beta	sig

Bivariate Analyses					

Age	1.018	(0.997–1.038)	0.09	0.087	0.104
Duration of diabetes	1.058	(1.026–1.091)	0	0.128	0.018
BMI	0.984	(0.942–1.027)	0.449	-0.009	0.865
SBP	1.006	(0.995–1.017)	0.286	0.094	0.082
DBP	1.012	(0.993–1.032)	0.209	0.075	0.163
Cholesterol	0.998	(0.995–1.001)	0.197	-0.066	0.207
Triglycerides	1.002	(1–1.003)	0.058	-0.012	0.819
HDL-C	0.984	(0.97–0.998)	0.03	-0.148	0.005
APOB	0.998	(0.992–1.005)	0.641	-0.032	0.542
LDL-C	0.996	(0.991–1.001)	0.115	-0.033	0.548
Lp(a)	1.002	(0.997–1.007)	0.423	0.063	0.233
HbA1C	1.106	(0.975–1.254)	0.118	0.239	0.008
GFR	1.003	(0.998–1.009)	0.229	-0.119	0.062
Sex (referent: male)	0.501	(0.323–0.777)	0.002	-0.124	0.013
Smoking	2.602	(1.304–5.194)	0.007	0.03	0.605

Multivariate Analyses	Model 1				

Sex	0.618	(0.367–1.041)	0.07	-0.057	0.31
Duration of diabetes	1.065	(1.03–1.101)	0	0.156	0.005
HDL-C	0.978	(0.962–0.995)	0.012	-0.176	0.002

	Model 2				

Sex	0.526	(0.193–1.433)	.209	-0.105	0.261
Duration of diabetes	1.060	(1.005–1.119)	.032	0.132	0.150
HDL-C	0.961	(0.929–0.994)	.021	-0.255	0.007
HbA1c	1.200	(1.028–1.4)	.021	0.261	0.005

Data are Exp B (confidence intervals), Beta Standardized Coefficients, and p values (sig).

BMI:body mass index;SBP: systolic blood pressure; DBP: diastolic blood pressure; GFR: glomerular filtration rate

Predictors of Model 1: HDL-C, duration of diabetes (yrs), and sex; Predictors of Model 2: HbA1C, HDL-C, duration of diabetes(yr), and sex

## Discussion

This study showed a relatively high prevalence of albuminuria (40%) in a population of Iranian type 2 diabetic patients who had an average duration of diabetes of 9 years. However, men had higher mean value of UAE compared to women, the age and HDL-C adjusted UAE levels of men and women were not significantly different. Albuminuria was significantly associated with the duration of diabetes in patients. HDL-C levels were lower among subjects with increased UAE and UAE was higher among those with uncontrolled glycemia (indicated by higher HbA1c levels).

The prevalence of albuminuria in diabetic patients of this study is in accordance with other reported cumulative incidence of albuminuria 5 to 10 years after diagnosis of T2D in other studies [13,14]. It has been clearly demonstrated that cumulative incidence or prevalence of microalbuminuria and renal impairment is associated with a longer duration of diabetes [15]. Despite the higher mean level of unadjusted UAE in men as compared to women, the difference in age and HDL-C adjusted UAE values of males and females was not statistically significant. Such a difference in unadjusted UAE values between men and women could be simply attributed to different prevalence of other cardiovascular risk factors between the two genders. This result support the idea that men and women with similar levels of cardiovascular risk factors may be expected to have similar risk of albuminuria. This has been shown by some prospective studies reporting that male sex does not increase the risk of progression to albuminuria, independent of the effect of other predictors [16-21], but is in contrast with the findings of PREVEND study which concluded that gender differences existed in the association between cardiovascular risk factors and UAE [22].

Molich et al has exhibited the association of lower HDL level and UAE in patients with long standing type 1 diabetes [23]. This study confirms such an association in subjects with type 2 diabetes. Increased UAE in patients with dyslipidemia may be secondary to dyslipidemia associated endovascular damage [24-27]. In this regard, there is some evidence that lipid reduction by antilipemic agents might decrease proteinuria in diabetic patients [28,29]; however, presence of direct causal correlation between dyslipidemia and diabetic renal damage is still a subject of controversy [30,31].

This study suffers from some limitations. Firstly, the UAE was measured once which may obscure conclusions on persistent albuminuria and the risk of progression to incipient or overt diabetic renal damage due to fluctuations in UAE in the course of disease. This is however a limitation of any cross-sectional study in this field. Secondly, we did not collect certain important variables such as diet, anemia, and drug history which might differ substantially between genders and may impact on the results. However, ignored confounding factors are more likely to cause false positive correlations rather than false negative results. Furthermore, we did not collect the history of cardiovascular diseases in our patients. Thirdly, HbA1c was integrated into our study for the final few month of the study period.

## Conclusion

We conclude that HDL-C and duration of diabetes are associated with increased UAE. Abnormal UAE is more frequent in men but this higher frequency may be explained by higher other cardiovascular risk factors in male gender.

## Competing interests

The authors declare that they have no competing interests.

## Authors' contributions

MN conceived and designed the study, provided expertise and oversight throughout the process, and participated in data collection. AE, FE, and SH assisted in data interpretation. NA carried out data collection. AM designed and participated in performing statistical analyses. MA conducted the statistical analyses and literature review, and drafted the manuscript. All authors read and approved of the final manuscript.
